# Reducing complexity in an agent based reaction model—Benefits and limitations of simplifications in relation to run time and system level output

**DOI:** 10.1016/j.biosystems.2016.06.002

**Published:** 2016-09

**Authors:** David M. Rhodes, Mike Holcombe, Eva E Qwarnstrom

**Affiliations:** a Department of Cardiovascular Science, Medical School, University of Sheffield, Sheffield, S10 2RX, United Kingdom; b Department of Computer Science, University of Sheffield, Sheffield, S1 4DP, United Kingdom

**Keywords:** Agent-based computational model, Complexity, Limitations, Scale, Iterations, Runtime, Model reduction

## Abstract

Agent based modelling is a methodology for simulating a variety of systems across a broad spectrum of fields. However, due to the complexity of the systems it is often impossible or impractical to model them at a one to one scale. In this paper we use a simple reaction rate model implemented using the FLAME framework to test the impact of common methods for reducing model complexity such as reducing scale, increasing iteration duration and reducing message overheads. We demonstrate that such approaches can have significant impact on simulation runtime albeit with increasing risk of aberrant system behaviour and errors, as the complexity of the model is reduced.

## 1 Introduction

Agent based modelling (ABM) is a methodology (Niazi and Hussain, 2011) for modelling complex systems used in a large variety of scientific fields from engineering and manufacturing to biology, ecology and social sciences (Coakley et al., 2006, Deissenberg et al., 2008, Holcombe et al., 2012, Grimm and Railsback, 2005, Shen et al., 2006, Macy and Willer, 2002). ABM uses a bottom up approach where agents are autonomous entities representing individual components of the system being modelled and system level behaviour is an emergent property of the actions and interactions of the various agents. However in many cases the systems being modelled may be comprised of millions or potentially billions of components and a model faithfully incorporating each individual as an agent may be either impossible to run on current hardware or have a runtime or volume of output data that make simulations impractical to perform. In these cases using ABM requires greater levels of abstraction to be employed within the model in order for it to be practical. Marked abstractions can be justified in cases where system behaviour is the focus of the analysis and the status of individual agents is not central to the output of the simulation. Hence as long as the abstraction is limited, so that the model is unaltered at the level of overall functionality, reduced complexity simulations still provide useful outputs.

Here we use a simple ABM based on a chemical reaction as described previously (Andrews and Bray, 2004, Pogson et al., 2006), and which has recently been expanded upon to explore specific aspects of biological regulatory systems (Pogson et al., 2008, Rhodes and Smith et al., 2015), to assess the impact of reducing model complexity on performance and system level output. The aims were to determine the degree to which simulations could be simplified without losing system level output while maintaining the core system design.

## 2 Material and methods

### 2.1 Agent based model

**Fig. 1:**
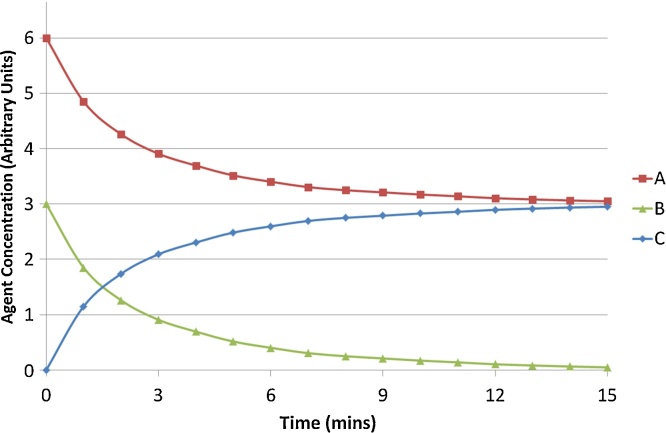
Agent concentrations in a typical simulation. Concentration of Agents A,B and C over time in a typical simulation run for the reaction A + B −> C.

To test the impact of varying settings within a simulation we used a simple model of a reaction between two different types of agents which combine to form a third in a one to one ratio (A + B −> C). The agents move within a bounded volume of space by a random walk implementation of Brownian motion and will interact with each other within a given range based on their affinity and the simulation settings (Pogson et al., 2006, Pogson et al., 2008). Fig. 1 demonstrates the output of a typical simulation using a starting concentration of A which is 2 times that of B. This simple reaction model was used in favour of a more complex system for the tests for several reasons. Firstly the reaction is a single step process involving only one interaction between agents which allows evaluation by simply measuring agent population levels. While in a more complex system in which there are more reactions or multiple reactions per agent, changes/errors in system behaviour could be masked through feedback mechanisms. In addition, agent based models are based on the idea of system level behaviour emerging from many low level interactions such as this reaction. If complexity changes do not impact the behaviour of the low level interactions then the emergent behavior should remain the same independent of the number of low level interactions within the system.

The ABM was developed in FLAME (Greenough et al., 2008) a platform designed for high performance and parallel processing of agents. In FLAME agents are modelled as state machines with memory, using transition functions between states which can read and write to the agent’s memory. Communication in FLAME is achieved entirely by messages which can by input and output by transition functions and are available to all agents simultaneously. In order to maintain synchronicity a function that needs to read a message must wait until all message outputs from all agents have been completed before it can begin. This synchronous messaging system allows agents to be processed in parallel across multiple processors and eliminates the impact of the order in which agents are processed on the behaviour of the model.

### 2.2 Model complexity reduction

#### 2.2.1 Scale

The scale of the simulation (the number of agents) was adjusted over a range of 2000 fold to a level at which the system no longer accurately represented the behaviour of the complete population. The reduction in the number of agents is compensated by a proportional increase in their interaction volumes. As interactions take place within a 3-D environment, a fixed distance interaction range produces a sphere volume of space around the agent in within which interactions can occur. Hence as the volume of a sphere is proportional to the cube of its radius, the interaction range is modified by the cube-root of the scale change according to the formula:

range = baseRange × (∛(1/modelScale))

Therefore if the population is halved, the volume of the sphere of interaction range is doubled. The sphere of interaction determines the rate at which agents can interact and so in this simulation is acting as a rate constant modifier. The rate of this particular reaction is dependent on the rate constant and concentrations of both reagent agents and hence halving both concentrations (by reducing the scale by half) would result in a reduction in reaction rate to one quarter without modifying the rate constant. By changing the interaction range to double the rate constant while halving both agent concentrations the overall reaction rate is halved with the aim of having the percentage of agents reacting over a given time remaining the same.

#### 2.2.2 Time step

To assess the impact of less frequent updating and consequent loss of detail on system behaviour, the length of each iteration was increased. Agent behaviour was maintained by proportionally changing the movement and interaction volume of each agent, with a doubling of the time step resulting in a doubling of the interaction volume according to the formula:

range = baseRange × (∛(timeStep))

In this case an increase in interaction range adjusts the rate constant to increase the effective reaction rate per iteration to compensate for a reduced number of iterations per simulation. The reaction rate measured per second should however remain the same as each iteration represents a longer period of time.

Agent movement uses a random walk implementation of Brownian motion with the length of each random step governed by a diffusion coefficient. Hence the change in size of each random step is proportional to the root of the change in time step according to the formula:

distance = (√(diffusion × timestep))

Table 1 shows the parameters used in the reaction model. The simulation environment was modelled as a simple sphere with agents bound within its volume (derived from the environment radius parameter) and remaining the same size throughout the tests. The numbers of agents in the starting iteration was adjusted over a range of 2000 fold to adjust the scale of the simulation with the interaction range adjusted as previously described. The time step was increased up to 600 fold with interaction volume increased proportionately. The diffusion coefficient remained constant throughout all tests while the distance of each random walk step varied with time step, as described above.

Simulation starting states used agents generated in random positions throughout the simulation environment so that each run tested a unique set of interactions between agents. To determine if complexity reductions increased the variance of the system level output produced by these random starting states, each test was repeated 5 times.

### 2.3 Agent messaging

Agent based models rely on the ability of agents to communicate with each other in order for complex behaviour to emerge. Agent interactions can produce conflicts that need to be resolved for example when multiple agents attempt to interact with a single other agent. Depending on the complexity of the conflict resolution system implemented in the model errors in interactions can occur. Next simulations were designed to investigate the overheads and impact of conflict resolution within the broadcast messaging system of the FLAME framework. In this framework agents communicate entirely through messages, which can be sent and received into their local vicinity. Messages can contain general information such as a location broadcast or be designated for a specific agent such as a request for interaction. This broadcast message system is one of many types used in ABM however all systems need the ability for agents to communicate and that communication makes up part of each iteration’s computational overhead.

Illustration of results of interaction checks performed using varying systems. No messaging (A) can result in loss or duplication of agents. Interaction confirmation (B) can solve the loss or duplication errors but can miss potential interactions that can occur (C). Looping interaction confirmations (D), show how the second loop of checks picks up the interactions missed in the initial interaction confirmation check.

#### 2.3.1 No messaging

**Fig. 2:**
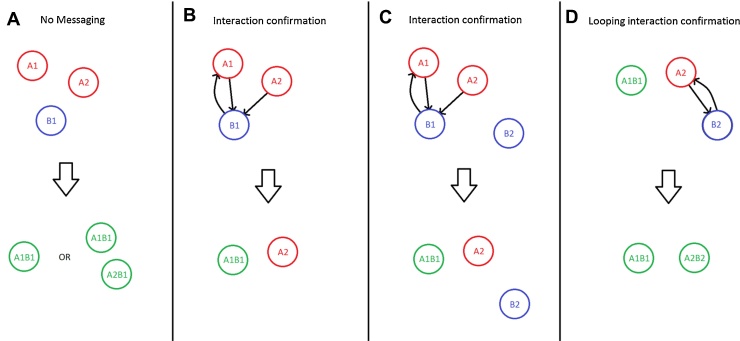
Agent interaction conflict resolution systems.

If an agent is within interaction range with another it assumes interaction occurs with no communication between the agents. If more than one agent is within interaction range with another they will both assume reaction has occurred and alter state, generating errors within the simulation. In the example in Fig. 2A two agents of type A assume binding with one agent of type B, depending on the implementation of the interaction this will lead to either an overall loss of one type A agent and the formation of one type AB or the overall creation of a duplicate type B agent due to the formation of two type AB agents.

#### 2.3.2 Interaction confirmation

One agent type sends requests for interaction to the nearest interacting agent within range and the responding agent sends out a single message to confirm the reaction can occur to the closest agent it received a request from. This process will eliminate the loss/duplication errors that occur with no messaging, therefore if two agents both request interaction with the same agent, that agent will respond to only one with a confirmation, illustrated in Fig. 2B. However as in Fig. 2C if an agent (A2) requests an interaction and is denied due to a closer agent being confirmed a potential interaction with a more distant agent (A2 and B2) can be missed leading to errors from lost interactions.

#### 2.3.3 Looping interaction confirmation

Messaging occurs in the same way as with Interaction confirmation (Fig. 2C) but repeats the cycle of messages until all possible interactions have occurred (Fig. 2D). This eliminates both error from loss/duplication and error from lost interactions but at the cost of increased computing overhead.

We tested the impact of having no messaging versus a full looping confirmation system in terms or both runtime and duplication error rate under varying density settings. For each density we adjusted the volume of the simulation environment while maintaining total agent numbers hence forcing the agents into higher or lower concentrations and therefore increasing the likelihood of creating an interaction conflict.

## 3 Results and discussion

### 3.1 Scale and time step

**Fig. 3:**
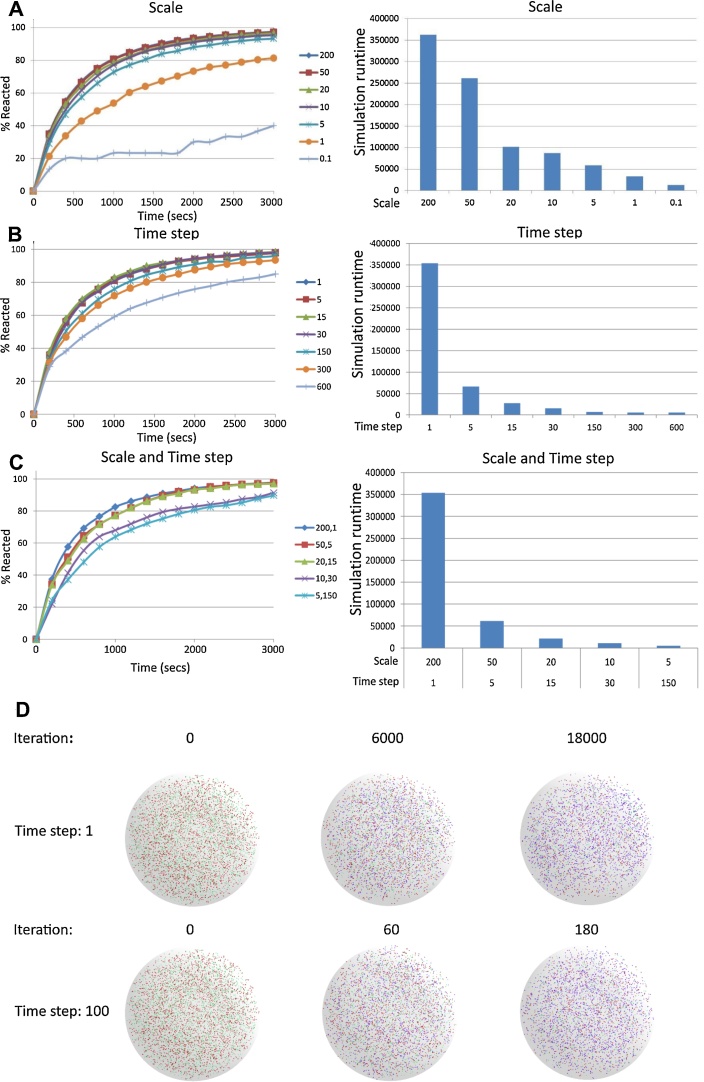
Impact of adjusting scale and time step on simulation.

From the results shown in Fig. 3A, the simulations in which the scale of the model was reduced, the adjustments to interaction range adjusting the rate constant of the reaction appear to compensate for the variation in agent concentrations. The results demonstrate that the overall system behaviour remained largely unchanged across several fold reduction in model scale. However, as the reductions became more extreme (2000 fold, less than 100 total agents) the system behaviour began to deviate noticeably and eventually break down. Reducing the scale and the total number of agents in the simulation lowers the number of agent interactions to be processed per iteration, reducing runtime. Similarly as the data produced per iteration includes all agents, the size of the simulation output files was proportionally reduced.

We see a similar impact on system level output when increasing iteration time steps, leaving the output consistent across alteration of several fold but diverging at extreme changes (600 fold) (Fig. 3B). Increasing the length of each time step reduces the total number of iterations required and results in a decrease in the overall runtime, which is roughly proportional for the majority of increments. However a minimum simulation runtime overhead appears to remain at the extreme time steps, preventing any further reduction in runtime. As the number of iterations processed is reduced proportionally to the time step, so is the amount of simulation data output. As agent movement in this simulation uses a random walk implementation, each agent moves in random jumps in each iteration, as the length of iterations increase so does the length of the jump. At very large time-steps agents may traverse a large proportion on the simulation environment in a single iteration. As the reaction model in this case was designed with an empty environment, large distance steps had little consequence on the simulation with the agent distribution remaining spread throughout the simulation volume throughout the tests (Fig. 3D). However in a simulation with physical obstacles, large distance time-steps may impact agent movement leading to noticeable divergence in system behaviour.

Combining both time step increases and scale reductions showed similar overall behaviour, but with divergence presenting earlier (20 fold time step and 30 fold scale) when combined than by changing either parameter alone (Fig. 3C). In addition, runtime reductions were slightly greater when combining changes in time step and scale, demonstrating that effects on runtime improvements and simulation divergence were additive.

Despite the system level output varying due to changes of scale and time-step, the variance between individual runs at any setting remained highly consistent despite the random starting locations of each agent. At the highest scale the standard deviation (SD) over 5 runs was 0.0004 at the end time point, while reducing the scale by 100 fold only increased the SD to 0.0041. Further halving in scale to the point where the simulation broke down did increase the variance to 0.0296. Similarly an increase in time-step by 600 fold changed the SD from 0.0041 to 0.0082. This demonstrates that up to the point where simulation behaviour breaks down these complexity changes do not make the variance between runs significantly higher and accurate readings can be obtained by few simulation run repeats. (A) Scale of simulation was adjusted by varying agent numbers by indicated proportion and interaction volumes by the inverse amount. Time step was fixed to 1 in each case. (B) Time step for each iteration was varied, increasing movement and interaction volumes by indicated amount and reducing total number of iterations by the same proportion. Scale was fixed to 200 in each case. (C) Scale and time step were varied simultaneously by indicated amounts. Graphs display percentage of total number of agent type A reacted (left) and total simulation time (right). (D) Agent distribution throughout simulation volume at different equivalent time points with time step adjusted 100 fold. Colours represent different agent types, Red − A, Green − B, Blue − C. Pictures are from a single simulation run for each time step setting, using the same initial agent distribution.

### 3.2 Agent messaging and density

**Fig. 4:**
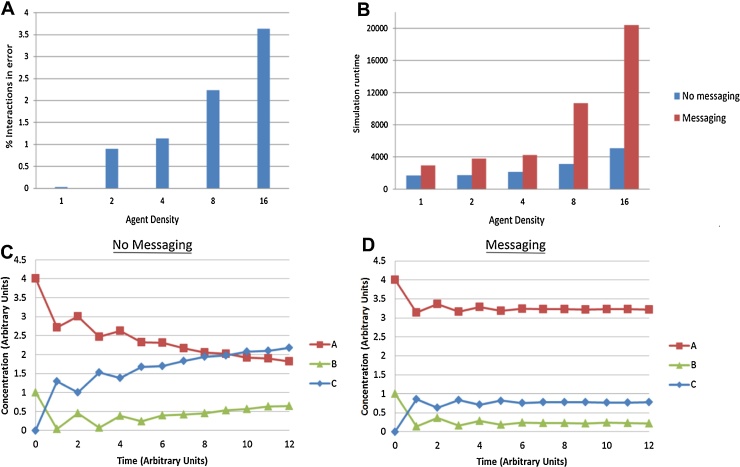
Impact of Messaging on Interaction Error and Runtime.

Forcing agents into higher concentrations by increasing density, increases the number of potential interactions in each iteration and thus increases the number of potential conflicts to be resolved. As can be seen in Fig. 4A, without a robust messaging system controlling potential interaction conflicts, a large number of erroneous interactions can occur, which may have a significant impact on system level behaviour. It can also be seen that conversely when interactions are much rarer, the likelihood of conflict is reduced and very few errors occur even without robust interaction checks. However from the runtime results in Fig. 4B we can see that the overhead of implementing the interaction confirmation loops is reasonably small when there are few conflicts to be resolved. At higher densities with larger number of conflicts, the message overhead becomes much higher resulting at the highest density with roughly 75% of the runtime being taken up by interaction resolution. Therefore we conclude that interaction confirmation is most costly on simulation runtime and although reducing the robustness of messaging would have a large impact on runtime it would also significantly increase error rate. In order to reduce both runtime by limiting messaging and error rate by minimizing interactions, the simulation density needs to be reduced to levels where interaction conflict is minimal and either the error rate is tolerable or the overhead of interaction confirmation is negligible. However, determining a tolerable error rate also depends on the nature of the interactions within the simulation. In some simulations interactions may feedback on themselves or from one another in a positive or negative manner. Negative feedback may reduce the impact of errors by self-regulating the system to compensate, whereas errors in positive feedback systems will compound leading to greater and greater error rates. An example of positive feedback can be seen in Fig. 4C in which the simple reaction model has had the addition of a disassociation mechanic which reverts agent C back to agents A and B after a fixed delay. In this system which does not include messaging, interaction errors cause duplication of agents as illustrated in Fig. 2, after dissociation these additional agents can rebind causing further errors. The repeated binding errors generate increasing numbers of erroneous agents leading to creation of far more of agent C than should have been possible from the starting agent concentrations. With messaging enabled (Fig. 4D) the system does not produce these errors and stabilizes at equilibrium between binding and dissociation. The difference between messaging systems in this model demonstrates the low tolerance for errors and the large changes in system output resulting from such positive feedback systems.

In the investigation of scale and time step we explored two methods of reducing simulation density which may help reduce this messaging overhead. Decreasing the simulation scale reduces the total number of agents and therefore the number or interactions occurring in each iteration, lowering the message system overhead in addition to the previously described runtime reductions. However the likelihood of conflict per agent will remain unchanged leading to errors without interaction confirmation. Reducing the time step of each iteration spreads interactions over multiple time points decreasing the effective density of the simulation and the likelihood of conflict. Reducing time step sufficiently will reduce error rates to a low enough level that message confirmation can be removed in favour of performance. However, reducing time steps increases the total number of iterations and hence runtime as previously shown, and may not result in an overall performance increase. Hence with some testing of tradeoffs between scale, time step, message overhead and error rate, runtimes and data output can be optimized while maintaining acceptable accuracy of system level output.

Error rate as percentage of total number of interactions resulting in agent duplications at varying simulation density (A) Runtime for simulations of varying density with no messaging or full confirmation loop messaging (B), results show messaging overhead correlates with number of interaction conflicts to resolve. Agent concentration in the simple reaction model with addition of dissociation of reacted agents, using no messaging (C) or full confirmation loop messaging (D). Duplication errors occurring without messaging can feedback resulting in increasing error over time whereas with messaging the system reaches equilibrium between binding and dissociation.

### 3.3 NFκB signaling pathway model

**Fig. 5:**
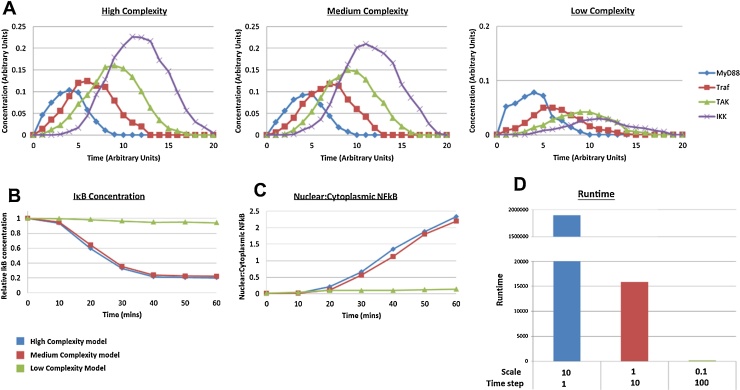
Impact of complexity on NFκB model.

To determine if these modifications could be applied to more complex models we tested scale and timestep changes on the ABM model developed as part of our previous work (Rhodes and Smith et al., 2015). This model is a cellular biological simulation of activation of the NFκB signaling pathway which contains multiple types of protein and receptor agents. The model features several types of interactions including binding, dissociation, transport, changes of state, agent destruction and creation and hence should be a more rigorous test of the complexity changes. Simulations were run in this model at three complexity levels, with the higher complexity model simulating approximately a 1:1 ratio of agent:protein and 1 s time steps. The medium complexity model was implemented with 10 fold-reduced scale, 10 fold increased time step and message confirmation present but limited to only the densest of agent interactions and the lowest complexity test was performed by reducing scale and increasing timestep by a further 10 fold. The results shown in Fig. 5A demonstrate the activation of several proteins within the signal pathway that activate each other in a cascade starting with MyD88 activation by a cell surface receptor. The high and medium complexity simulations show signal amplification through the cascade with similar peaks of activity in terms of both time and intensity. However the low intensity model simulations resulted in progressive dampening of activation through the pathway, demonstrating a significant reduction in the number of agent interactions occurring in a manner similar to that of the reaction rate tests. In Fig. 5B and C can be seen two typical system level outputs of the simulation, in which both the medium and the high complexity simulations agree well with *in vitro* data (Yang et al., 2003, Carlotti et al., 1999). The low complexity model however shows only a small change in these outputs, showing that the signal dampening effects of the complexity changes lead to almost no change at the bottom of the signal pathway. The reduction in complexity from high to medium resulted in a reduction in running time to less than 1/100th of the time required for the more complex model.

The results demonstrate the ability to significantly improve simulation performance without losing system level behaviour. We believe that through complexity changes such as these, improvements to runtimes can be achieved in a variety of ABMs, especially those with large numbers of relatively simple agents such as signalling pathways, cellular models (Kaul et al., 2013) and population models (Mathevet et al., 2003). Because of inherent differences in agent behaviour, interactions and environment between different ABM models, it is difficult to derive general rules regarding the effect of complexity on ABM output. However our data show that reduction in complexity may be a valuable consideration in design or optimisation specifically of large scale ABMs.

Protein activation cascade shows consistent amplification through the signal pathway at high and medium complexity but a dampening of signal at low complexity (A). Simulated degradation of IκB (B) and ratio of nuclear to cytoplasmic NFκB (C) over 60 min of stimulation with the cytokine interleukin1- (IL-1), are consistent between high complexity (blue) and medium complexity (red) models, but greatly reduced in low complexity simulations (green). Total simulation runtime (D) reduced over 100 fold from high to medium complexity and over a further 100 fold from medium to low complexity.

## 4 Conclusions

We have used a simple chemical reaction ABM to study the impact of reducing simulation complexity on the system level output and simulation runtime. We have shown the number of agents and the length of time step can be varied by several orders of magnitude and still produce very similar system level behaviour while having a large impact on runtime and data output. We also show that a lack of robust interaction messaging can lead to large error rates and yet the overhead of those robust checks can be significant. Therefore engineering the simulation in a way that generates less interaction conflict can provide large benefits in accuracy and/or runtime.

ABMs are rarely expected to produce perfect predictions, but are accepted as abstractions and used to identify trends in behaviour of the simulated system under specific conditions. Therefore the benefits of these complexity reductions in terms of runtime and data output may well outweigh any small inaccuracies in the simulation output.

## Figures and Tables

**Table 1:** Simulation parameters.

Variable	Formulae Symbol	Value at Model Scale 1 and Timestep 0.1
Environment radius		17.5 μm
Model Scale	modelScale	1
Diffusion Coefficient	diffusion	5 × 10^−5^ cm^2^/s
Timestep length	timestep	0.1 s
Interaction range (radius)	baseRange	0.3 μm
Starting Agent population − Agent A		300 × modelScale
Starting Agent population − Agent B		600 × modelScale
